# Enhanced status of inflammation and endothelial activation in subjects with familial hypercholesterolaemia and their related unaffected family members: a case control study

**DOI:** 10.1186/s12944-017-0470-1

**Published:** 2017-04-24

**Authors:** Thuhairah Rahman, Nur Suhana Hamzan, Atiqah Mokhsin, Radzi Rahmat, Zubin Othman Ibrahim, Rafezah Razali, Malathi Thevarajah, Hapizah Nawawi

**Affiliations:** 1 Faculty of Medicine, Universiti Teknologi MARA (UiTM), Jalan Hospital, Sungai Buloh, Selangor Malaysia; 2 Laboratory Medicine Division, Universiti Malaya Medical Center, Kuala Lumpur, Malaysia; 3 Institute of Pathology, Laboratory and Forensic Medicine (I-PPerForM), Universiti Teknologi MARA (UiTM), Sungai Buloh, Selangor Malaysia

**Keywords:** Coronary artery disease, Familial hypercholesterolaemia, Inflammation, Oxidative stress, Endothelial activation

## Abstract

**Background:**

Familial hypercholesterolaemia (FH) leads to premature coronary artery diseases (CAD) which pathophysiologically can be measured by inflammation, endothelial activation and oxidative stress status. However, the status of these biomarkers among related unaffected relatives of FH cases and whether FH is an independent predictor of these biomarkers have not been well established. Thus, this study aims to (1) compare the biomarkers of inflammation, endothelial activation and oxidative stress between patients with FH, their related unaffected relatives (RUC) and normolipaemic subjects (NC) (2)determine whether FH is an independent predictor of these biomarkers.

**Methods:**

One hundred thirty-one FH patients, 68 RUC and 214 matched NC were recruited. Fasting lipid profile, biomarkers of inflammation (hsCRP), endothelial activation (sICAM-1 and E-selectin) and oxidative stress [oxidized LDL (oxLDL), malondialdehyde (MDA) and F2-isoprostanes (ISP)] were analyzed and independent predictor was determined using binary logistic regression analysis.

**Results:**

hsCRP was higher in FH and RUC compared to NC (mean ± SD = 1.53 ± 1.24 mg/L and mean ± SD = 2.54 ± 2.30 vs 1.10 ± 0.89 mg/L, *p* < 0.05). sICAM-1 and E-selectin were higher in FH compared to NC (mean ± SD = 947 ± 742 vs 655 ± 191 ng/mL, *p* < 0.001 and 175 ± 131 vs 21.6 ± 10.7 ng/mL, *p* < 0.001 respectively) while sICAM-1 concentration was higher in RUC compared to NC (mean ± SD = 945 ± 379 vs 655 ± 191 ng/mL, *p* < 0.01). Biomarkers of oxidation (ox-LDL, MDA and ISP) were elevated in FH compared to NC [mean ± SD = (48.2 ± 26.8 vs 27.3 ± 13.2 mU/L, *p* < 0.001), (2.57 ± 1.3 vs 1.20 ± 0.30 nmol/mL, *p* < 0.001) and (645 ± 396 vs 398 ± 20.5 pg/L, *p* < 0.001) respectively], but no significant differences were observed between RUC and NC (*p* > 0.05). FH was an independent predictor for sICAM-1 (*p =* 0.007), ox-LDL (*p* < 0.001) and MDA (*p* < 0.001) while RUC independently predicted for sICAM-1 (*p* < 0.001).

**Conclusion:**

The screening for FH is vital as all biomarkers associated with atherogenesis are higher in these subjects and FH also independently predict biomarkers of endothelial activation and oxidative stress. Furthermore, despite not fulfilling the diagnostic criteria for FH, related unaffected family members that may not phenotypically express the mutation may still be at risk of developing CAD as reflected from the enhanced inflammatory and endothelial activation status observed in this group. This highlights the need to not only conduct family tracing in indexed FH cases, but also assess the coronary risk among family members that do not fulfil the FH diagnostic criteria.

## Background

Atherosclerosis has been well established to be a chronic inflammatory response involving a complex process where lipids, inflammatory and haemostatic components orchestrate plaque formation and progression with subsequent thrombus formation leading to coronary artery disease (CAD) such as myocardial infarction and ischaemic strokes. Several proinflammatory cytokines are responsible for the activation and dysfunction of vascular endothelium which leads to the overexpression of adhesion molecules [1], enhanced oxidized low density lipoprotein (oxLDL) uptake by monocyte-derived macrophages by increasing the expression of scavenger receptors on the cells surface [2] to form foam cells. Expression of inflammatory factors such as C-reactive protein (CRP), proinflammatory cytokines including interleukin-1 (IL-1) and interleukin-6 (IL-6), and adhesion molecules soluble intercellular adhesion molecule-1 (sICAM-1) and soluble vascular adhesion molecule-1 (sVCAM-1), establishes a strong relationship between inflammation and atherogenesis.

Atherosclerosis may be present throughout the lifetime of an individual. Fatty streaks, which represent the earliest sign of atherosclerosis, have been observed in fetal aortas and in children as young as 3 years of age [3–5]. To determine the degree of atherosclerosis, several surrogate biomarkers have evolved to represent the major characteristics of atherogenesis, namely endothelial activation, inflammation, oxidative stress and prothrombogenesis. Biomarkers reflecting endothelial activation such as intercellular adhesion molecules-1 (ICAM-1) [6], inflammation such as interleukin-6 (IL-6) and high sensitivity C-reactive protein (hsCRP) [6], oxidative stress such as malondialdehyde (MDA), oxidized LDL (oxLDL) [7] and F2-isoprostanes (ISP) [8] have been used to predict coronary risk and in the prognostication among patients with established atherosclerosis.

Familial hypercholesterolaemia (FH) is a genetic mutation of autosomal dominant inheritance which affect genes encoding for low-density lipoprotein receptor (LDL-R), proprotein convertase subtilisin/kexin type 9 (PCSK9) or apolipoprotein B (apoB) resulting in marked elevation of LDL cholesterol (LDL-c) concentrations [9]. Heterozygous forms of FH have inherited one mutation from one parent and are characterized by a 2-fold increase in both plasma total cholesterol (TC) and LDL-c concentrations [3, 10, 11]. It has been well established that patients with FH have higher risk of developing CAD compared to those with polygenic hypercholesterolaemia. Approximately 5% of patients suffering myocardial infarction below the age of 60 years have heterozygous FH [3, 10], which resorted to current guidelines determining FH patients as high risk for developing CAD without undergoing risk stratification algorithms such as the Framingham Risk Score as they do not predict the 10-year CHD risk in these cohort.

FH is one of the most common inherited disorders, with an estimated prevalence of 1 in 500, although the frequency may be higher in populations exhibiting founder effect [12, 13]. Compound heterozygotes have an incidence of 1 in 200,000 while that of homozygous is rare (approximately 1 in 1 million) [12]. Because FH patients are at greater risk of developing devastating atherosclerosis-related diseases, the need for early diagnosis becomes vital to ensure appropriate aggressive intervention is given to delay and possibly even prevent the progression of CAD.

It is well established that FH subjects have elevated concentrations of inflammatory, endothelial activation and oxidative stress biomarkers which puts them at risk of enhanced atherogenesis and possibly the development of premature CAD [14]. However, reports on whether FH is an independent predictor for these biomarkers, after correcting for confounding factors such as hypertension, diabetes mellitus, smoking and obesity, is limited.

Considering the high coronary risk these genetic mutations carry, guidelines have been established to diagnose patients with FH in order to identify positive cases early for appropriate management to prevent the onset of early complications such as premature CAD. Family tracing to identify affected family members have also been the cornerstone of early detection of FH among the first and second degree relatives. However, to date, there have not been extensive published data on the coronary risk status, by way of measuring biomarkers of inflammation, endothelial activation and oxidative stress, of unaffected family; and whether or not, despite not fulfilling the diagnostic criteria, they may still be at higher risk of CAD.

Therefore, the aim of this study was to investigate the status of inflammation, endothelial activation and oxidative stress in patients with FH and their related unaffected family members compared to normal controls and to identify the independent predictors of the biomarkers of inflammation, endothelial activation and oxidative stress after correcting for age, gender, hypertension, diabetes mellitus, smoking and body mass index status.

## Methods

### Recruitment of FH patients, RUC and NC groups

This was a cross-sectional, observational study involving 132 FH patients, 60 related, first-degree unaffected family members and 180 normolipaemic controls (NC). All subjects were screened through a protocol consisting of medical histories, physical examination and laboratory tests which included fasting plasma glucose, fasting serum lipid profile, renal profile, liver function and thyroid function profile which included thyroid stimulating hormone (TSH) and free thyroxine (fT4). Diagnosis of FH was made based on the Simon Broome Diagnostic Criteria [15]. Based on Simon Broome Diagnostic Criteria, diagnosis of Definite FH requires TC > 7.5 mmol/L or LDL-c > 4.9 mmol/L with at least one of two: 1) physical findings of tendon xanthomas, or tendon xanthomas in first or second degree relative. Possible FH is diagnosed based on TC > 7.5 mmol/L or LDL-c > 4.9 mmol/L with at least one of the two: 1) family history of myocardial infarction at age ≤ 60 years in first-degree relative or 2) myocardial infarction at age ≤ 50 years in second-degree relative.

Secondary causes of hypercholesterolaemia such as diabetes mellitus, nephrotic syndrome, and hypothyroidism were excluded. Upon diagnosis of FH case index, first-degree, unaffected relatives (RUC) were then recruited through cascade screening. Subjects with normal lipid profile (TC < 5.2 mmol/L, LDL < 4.1 mmol/L, TG < 1.7 mmol/L, HDL > 1.0 or 1.3 mmol/L in males and females respectively), non-diabetic, non-hypertensive, normal renal profile and liver function test were grouped as normolipaemic controls (NC). Exclusion criteria for all groups include recent febrile illness, concomitant neoplasm, inflammatory disease or immunosuppressive therapy including steroid usage and those taking anti-inflammatory agents or vitamin supplements were also excluded from this study. Venous blood samples were collected from the FH subjects upon diagnosis and prior to initiation of statins while all RUC were not on any medication.

### Topography measurements

Blood pressure (BP), body mass index (BMI), smoking habits and history of personal CAD and family history of premature CAD were documented. With the subject seated and after 5–10 min rest, BP was measured using an automated BP reader (cuff size 12 × 33 cm, Colin press-mate, Japan). The systolic blood pressure (SBP) and diastolic blood pressure (DBP) was measured to the nearest 1 mmHg. Height and weight was measured to obtain BMI by using the formula: BMI = weight (kg)/height^2^ (m^2^). Presence of CAD was assessed based on the clinical history, previous medical records and exercise tolerance test reports.

### Sample collection and analysis for biomarkers of inflammation, endothelial activation and oxidative stress

Blood samples were collected in the morning following 10 to 12 h of fasting. All blood samples were centrifuged at 3500 rpm for 7 min to extract serum and plasma samples which were kept frozen at −20 °C until laboratory testing at an ISO 15189:2007 accredited laboratory (SAMM 688). Fasting plasma glucose (FPG) was analyzed using the hexokinase method, while TC, triglyceride (TG), and HDL-c were measured by enzymatic reference methods, and hsCRP concentration was measured by turbidimetric assay. FPG, TC, TG, HDL-c and hsCRP were measured on an automated analyzer (Cobas Integra 400 PLUS, Roche Diagnostics, Germany). LDL-c concentration was derived using the Friedewald equation [16]. These tests were accredited by an international accreditation body (MS ISO 15189:2007, SAMM No. 688). Serum IL-6, sICAM-1 and E-selectin concentrations were measured by enzyme linked immunosorbent assay (ELISA) (eBioscience Bender MedSystems, Vienna Austria). Serum ox-LDL concentration was measured using an ELISA kit (Mercodia, Sweden). MDA concentration was measured by a method adapted from [17]. All absorbance were read on a microplate reader (Tecan Sapphire II, Austria). F2-isoprostanes concentration was analyzed by liquid chromatography-tandem mass spectrometry method on the 4000 QTRAP (Applied Biosystem, Canada) following pretreatment of the samples using diethyl ether.

### Statistical analysis

Demographic data were presented as mean ± SD for normally distributed variables or mean ± SD for non-normally distributed data, and as percentages for categorical data. Analysis of normality was performed using the Kolmogorov–Smirnov test. For normally distributed variables, comparisons between groups were performed using one way ANOVA. Categorical data and proportions were analysed using Chi-square test. Pearson’s or Spearman’s correlation coefficient was used to analyze correlations between two normal or non-normal distribution variables respectively. Binary logistic regression analysis was applied to determine independent determinant of the biomarkers after correcting for age, gender, ethnicity, HDL-c, diabetes, hypertension and smoking status. A *p* value of <0.05 was considered statistically significant. The statistical analysis was performed on the Statistical Package for Social Sciences (SPSS version 20.0).

## Results

### Demographic and clinical characteristics of study population

The demographic and clinical characteristics of the studied subjects are shown in Table 1. Compared to NC, FH patients had significantly higher SBP, TC, TG, LDL-c and HDL-c concentrations. There were no significant differences in BMI and DBP between the two groups. Both groups were matched for age, gender, ethnicity, smoking and hypertension status.

**Table 1:** Demographic Characteristic and concentration of inflammation, endothelial activation and oxidative stress biomarkers in FH, RUC and NC groups

Parameters	FH (*n* = 131)	RUC (*n* = 68)	NC (*n* = 214)	*p*-value (FH vs. NC)	*p*-value (FH vs. RUC)	*p*-value (RUC vs. NC)
^a^Age (years)	45.1 ± 1.21	29.8 ± 3.00	43.6 ± 0.81	NS	***	***
^b^Gender (% Female/Male)	55.7/44.3	60.3/39.7	58.4/41.6	NS	NS	NS
^b^Ethnicity (% Malay/Chinese/Indian)	76.3/22.1/1.5	60.3/39.7/0.0	82.2/15.4/2.3	NS	*	***
^b^Current smoker (%)	13.0	1.5	19.2	NS	NS	NS
^a^BMI (kg/m^2^)	24.8 ± 0.44	25.8 ± 2.18	25.3 ± 0.33	NS	NS	NS
^a^Systolic BP (mmHg)	133 ± 1.99	120 ± 4.00	124 ± 1.31	***	NS	NS
^a^Diastolic BP (mmHg)	76.6 ± 1.03	77.4 ± 2.56	77.3 ± 0.91	NS	NS	NS
^a^TC(mmol/L)	8.43 ± 0.18	5.16 ± 0.40	5.44 ± 0,06	***	***	NS
^a^TG (mmol/L)	1.94 ± 0.15	1.12 ± 0.20	1.53 ± 0.06	*	NS	NS
^a^HDL-c (mmol/L)	1.28 ± 0.03	1.45 ± 0.26	1.47 ± 0.26	***	NS	NS
^a^LDL-c (mmol/L)	6.26 ± 0.19	3.21 ± 0.33	3.29 ± 0.05	***	***	NS
Biomarkers of inflammation, endothelial activation and oxidative stress						
^a^hsCRP (mg/L)	1.53 ± 1.24	2.54 ± 2.30	1.10 ± 0.89	*	***	***
^a^sICAM-1 (ng/mL)	947 ± 742	945 ± 379	655 ± 191	***	NS	**
^a^E-selectin (ng/mL)	175 ± 131	7.89 ± 4.05	21.6 ± 10.7	***	***	NS
^a^ox-LDL (mU/L)	48.2 ± 26.8	25.2 ± 20.6	27.3 ± 13.2	***	***	NS
^a^MDA (nmol/mL)	2.57 ± 1.30	2.0 ± 0.9	1.20 ± 0.30	***	***	***
^a^F2- Isoprostanes (pg/L)	645 ± 396	NA	398 ± 20.5	***	-	-

*FH* familial hypercholesterolaemia, *RUC* related unaffected controls, *NC* normolipaemic subjects *BMI* body mass index, *BP* blood pressure, *TC* total cholesterol, *TG* triglycerides, *HDL-c* high density lipoprotein cholesterol, *LDL-c* low density lipoprotein cholesterol, *hsCRP* high sensitivity C-reactive protein, *sICAM-1* soluble intercellular adhesion molecule-1, *MDA* Malondialdehyde

Data were expressed as ^a^mean ± SD, or ^b^percentage. *p < 0.05, **p < 0.01, ***p < 0.001, NS: not significant

RUC group had no statistical difference compared to NC with regards to gender, smoking status, BMI, SBP, DBP, diabetic status, TC, TG, HDL-c and LDL-c concentrations; except for age, ethnicity and hypertension status.

### Comparison of concentrations of biomarkers between FH, RUC and NC groups

**Fig. 1:**
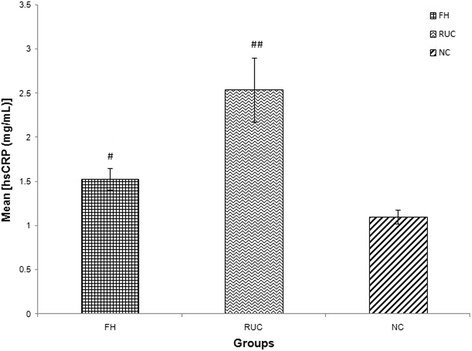
Graph of mean concentration of hsCRP in three different groups. Data were expressed as mean ± SEM. FH and RUC groups were higher in hsCRP concentration compared to NC. ## indicated *p* < 0.001 and # *p* < 0.05 compared to NC; FH: Familial Hypercholesterolaemia; RUC: Related unaffected controls; NC: normolipaemic subjects

**Fig. 2:**
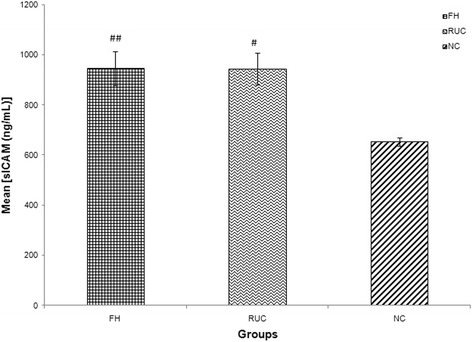
Graph of mean concentration of sICAM-1 in three different groups. Data were expressed as mean ± SEM. FH and RUC groups were higher in sICAM-1 concentration compared to NC. ## indicated *p* < 0.001 and # *p* < 0.01 compared to NC; FH: Familial Hypercholesterolaemia; RUC: Related unaffected controls; NC: normolipaemic subjects

**Fig. 3:**
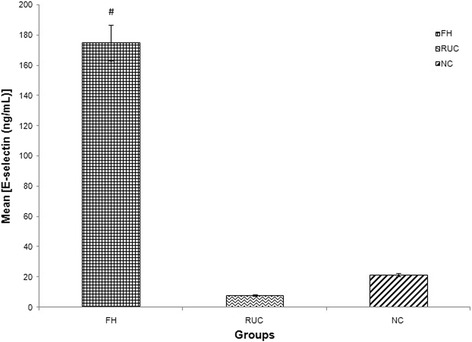
Graph of mean concentration of E-selectin in three different groups. Data were expressed as mean ± SEM. FH group was higher in E-selectin concentration compared to NC. # indicated *p* < 0.001 compared to NC; FH: Familial Hypercholesterolaemia; RUC: Related unaffected controls; NC: normolipaemic subjects

**Fig. 4:**
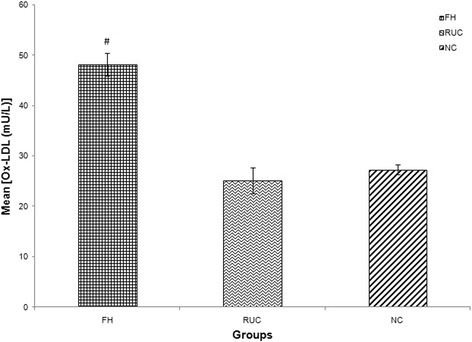
Graph of mean concentration of Ox-LDL in three different groups. Data were expressed as mean ± SEM. FH group was higher in Ox-LDL concentration compared to NC # indicated *p* < 0.001 compared to NC; FH: Familial Hypercholesterolaemia; RUC: Related unaffected controls; NC: normolipaemic subjects.

**Fig. 5:**
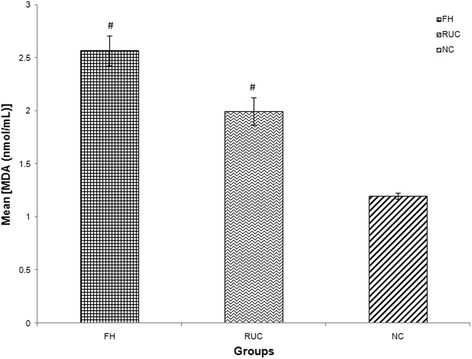
Graph of mean concentration of MDA in three different groups. Data were expressed as mean ± SEM. FH and RUC groups were higher in MDA concentration compared to NC. # indicated *p* < 0.001 compared to NC; FH: Familial Hypercholesterolaemia; RUC: Related unaffected controls; NC: normolipaemic subjects

**Fig. 6:**
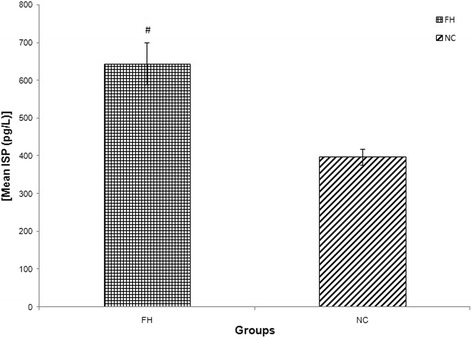
Graph of mean concentration of ISP in two different groups. Data were expressed as mean ± SEM. FH group was higher in ISP concentration compared to NC. # indicated *p* < 0.001 compared to NC; FH: Familial Hypercholesterolaemia; RUC: Related unaffected controls; NC: normolipaemic subjects

FH patients had higher concentration of inflammatory biomarker (hsCRP) compared to NC (mean ± SD = 1.53 ± 1.24 vs 1.10 ± 0.89 mg/L, *p* < 0.05). FH patients also exhibited a greater state of endothelial activation, as indicated by higher concentrations of sICAM-1 (mean ± SD = 947 ± 742 vs 655 ± 191 ng/mL, *p* < 0.001) and E-selectin (mean ± SD = 175 ± 131 vs 21.6 ± 10.7 ng/mL, *p* < 0.001) compared to NC. Oxidative stress biomarkers such as ox-LDL, MDA and ISP concentrations were higher in FH when compared to NC; [mean ± SD = (48.2 ± 26.8 vs 27.3 ± 13.2 mU/L, *p* < 0.001), (2.57 ± 1.3 vs 1.20 ± 0.30 nmol/mL, *p* < 0.001) and (645 ± 396 vs 398 ± 20.5 pg/L, *p* < 0.001) respectively]. RUC had significantly higher hsCRP concentration compared to NC (mean ± SD = 2.54 ± 2.30 vs 1.10 ± 0.89 mg/L, *p* < 0.001). With regards to endothelial activation status, RUC had higher sICAM-1 concentration than NC (mean ± SD = 945 ± 379 vs 655 ± 191 ng/mL, *p* < 0.01) while there was no significant difference in E-selectin concentration compared to NC (mean ± SD = 7.89 ± 4.05 vs 21.6 ± 10.7 ng/mL, *p* > 0.05). No significant differences were observed between RUC and NC for ox-LDL, (mean ± SD = 25.2 ± 20.6 vs 27.3 ± 13.2 mU/L, *p* > 0.05). The summary of these findings are tabulated in Table 1 and illustrated in Figs. 1, 2, 3, 4, 5 and 6.

### Association between FH, RUC and NC groups with concentration quartiles of the biomarkers

Table 2 summarizes the association between FH, RUC and NC groups with concentration quartiles of the biomarkers of interest by Chi square analysis. It showed FH, RUC and NC groups were inversely associated with the quartiles of all biomarkers. A majority of FH patients and RUC were found in the highest quartiles of each biomarker except for E-selectin in RUC group.

**Table 2:** Association between groups with quartiles of biomarkers of inflammation, endothelial activation and oxidative stress

Biomarkers	FH	RUC	NC	*p*-value
	n (%)	n (%)	n (%)	
hsCRP (mg/L)				
< 0.30	19 (18.3)	3 (7.5)	22 (15.3)	*0.017*
0.30–0.69	15 (14.4)	5 (12.5)	39 (27.1)	
0.70–1.49	25 (24.0)	11 (27.5)	42 (29.2)	
≥ 1.5	45 (43.3)	21 (52.5)	41 (28.5)	
sICAM-1 (ng/ml)				
< 472	54 (45.8)	3 (8.3)	20 (13.0)	*<0.001*
472–603	6 (5.1)	4 (11.1)	48 (31.2)	
604–772	7 (5.9)	6 (16.6)	50 (32.5)	
≥ 773	51 (43.2)	23 (63.9)	36 (23.3)	
E-selectin (ng/mL)				
< 16.53	8 (6.5)	50 (100)	65 (36.9)	*<0.001*
16.5–26.0	8 (6.5)	0	54 (30.2)	
26.1–101.7	29 (23.6)	0	59 (33.0)	
≥ 101.8	78 (63.4)	0	0	
ox-LDL (mU/L)				
< 23.9	27 (24.1)	38 (59.4)	71 (39.0)	*<0.001*
23.9–35.4	8 (7.1)	5 (7.8)	62 (34.1)	
35.5–53.7	26 (23.2)	15 (23.4)	43 (23.6)	
≥ 53.8	51 (45.5)	6 (9.4)	6 (3.3)	
MDA (nmol/mL)				
< 1.02	19 (22.1)	6 (12.5)	43 (27.4)	*<0.001*
1.02–1.37	2 (2.3)	5 (10.4)	71 (45.2)	
1.38–2.33	11 (12.8)	24 (50.0)	43 (27.4)	
≥ 2.34	54 (62.8)	13 (27.1)	0 (0.0)	
F2- Isoprostanes (pg/L)				
< 1.04	0 (0.0)		64 (42.1)	*<0.001*
1.04–3.48	0 (0.0)		47 (30.9)	
3.49–308	11 (21.6)		41 (27.0)	
≥ 309	40 (78.4)		0 (0.0)	

*FH* familial hypercholesterolaemia, *RUC* related unaffected controls, *NC* normolipaemic subjects, *hsCRP* high sensitivity C-reactive protein, *sICAM-1* soluble intercellular adhesion molecule-1, *MDA* Malondialdehyde

Italic *p* value < 0.05 consideredsignificant

### Independent predictor of biomarkers

To further explore the independent effect of FH and RUC on these biomarkers, binary logistic regression analyses were performed with the biomarkers as dependent variables. It was found that FH is an independent predictor for sICAM (*p =* 0.007), ox-LDL (*p* < 0.001) and MDA (*p* < 0.001) after correcting for age, gender, diabetes mellitus, hypertension, smoking status and BMI. RUC was found to be an independent predictor for sICAM (*p* < 0.001), after correcting for the same parameters. (See summary in Table 3).

**Table 3:** Independent predictors for endothelial activation and oxidative stress biomarkers in all subjects

Biomarkers	Independent predictors	Constant	Beta	SE	Adjusted OR	95% CI (Lower boundary, Upper boundary)	*p*-value
sICAM-1	FH	-0.98	0.669	0.247	1.95	1.20, 3.17	0.007
	RUC	-1.42	1.871	0.398	6.50	2.98, 14.17	<0.001
Ox-LDL	FH	-0.75	1.621	0.248	35.1	3.11, 8.23	<0.001
MDA	FH	-2.49	3.56	0.39	5.06	16.4, 75.3	<0.001

*sICAM* soluble intercellular adhesion molecule-1, *MDA* Malondialdehyde

The model reasonably fits well. Model assumptions are met. There are no interaction and multicollinearity problem

FH and RUC are independent predictors for sICAM after correcting for age, gender, ethnicity, HDL-c, diabetic, hypertension and smoking status

FH is an independent predictor for oxLDL and MDA after correcting for age, gender, ethnicity, HDL-c, diabetic, hypertension and smoking status

## Discussion

The significantly increased risk of CAD associated with FH was reported long before lipid lowering medications were widely prescribed [18–23]. There have been numerous studies since the 1990s that reported significant increases in biomarkers of inflammation, endothelial activation and oxidative stress in FH. This present cross-sectional study further substantiated those findings by demonstrating increases in serum hsCRP, sICAM-1, E-selectin, MDA, oxLDL and ISP concentrations among patients with FH, which reflect greater risk of developing premature CAD in these patients with a mean age of 45.1 ± 1.21 years. In addition, we further showed that related unaffected family members had higher inflammatory and endothelial activation status compared to normal healthy controls, suggesting increased coronary risk in these subjects despite not fulfilling the diagnostic criteria for definite or possible FH. These important findings possibly demonstrate the various genetic polymorphisms which translate to the enhancement of inflammatory, endothelial activation and oxidative stress status other than severe hypercholesterolaemia. Various complex interactions between genes and environmental factors may influence the variation of phenotypic expression from abnormal lipid concentrations to the development of apparent clinical disease [24, 25].

Furthermore, this present study clearly demonstrated that RUC was an independent predictor for sICAM-1 (*p* < 0.001) after correcting for the various confounding factors such as age, gender, hypertension, diabetes mellitus and BMI status. To the best of our knowledge, there are scarce reports determining these coronary risk biomarkers in unaffected relatives of FH subjects, particularly among the Asian population and whether related unaffected families are independent predictor of these biomarkers.

The initial step in the development of an atherosclerotic lesion involves dysfunction of the endothelial vessel wall. Endothelial dysfunction can be observed in the peripheral vessels of subjects with increased CAD risk as early as in the first decade of life [26]. A study by Guardamagna et al. (2009) reported significantly increased carotid intima media thickness and serum P-selectin levels among dyslipidaemic children compared to normal control, while serum hsCRP was higher in FH children compared to familial combined hyperlipidaemic children [27]. Animal studies have suggested that endothelial dysfunction may be caused by a variety of insults such as infection, smoking or elevated concentrations of lipoproteins such as LDL-c that triggers inflammation. [28] Once endothelial barrier becomes dysfunctional, LDL-c is able to enter the vessel wall and become oxidized, causing subsequent recruitment of monocytes from the blood. These cells differentiate into macrophages and engulf the oxidized LDL-c to form lipid laden foam cells [26] which are then deposited as cholesterol crystals within the tunica intima [29]. sICAM-1 is an endothelial- and leukocyte-associated transmembrane protein long known for its importance in stabilizing cell-cell interactions and facilitating leukocyte endothelial transmigration. Activated endothelium releases soluble adhesion molecules and therefore, measurement of fluid-phase adhesion molecules is used to quantify endothelial activation. Similarly, E-selectin is a carbohydrate-binding molecule found in endothelial cells, where during an inflammatory response, is responsible for the attachment and slow rolling of leucocytes along the vascular wall [30, 31]. There is also evidence to suggest that selectin engagement can trigger signaling events within endothelial cells through E-selectin [32]. These findings implicate selectin as biomarkers of endothelial activation.

This study illustrated higher serum concentrations of sICAM-1 and E-selectin in FH patients compared to matched normal control subjects. Van Haelst et al. [6] demonstrated similar findings of elevated ICAM-1 levels in FH subjects whilst [33] have reported a strong association between increased sICAM-1 levels in FH children and impaired endothelial function. Another research studied levels of sICAM-1 and sVCAM-1 in patients with familial combined hyperlipidaemia (FCH) and found that asymptomatic members of FCH families have higher plasma concentrations of these biomarkers than controls. However, their normolipaemic relatives exhibited no significant differences either of sVCAM-1 or of sICAM-1 concentrations [34]. This is in contrast to findings of this study which showed significantly higher concentration of sICAM-1 in related unaffected family members compared to normal controls, where confounding factors such as hypertension, glucose intolerance and obesity have been excluded. This strongly suggests that despite not having hypercholesterolaemia fulfilling any of the FH diagnostic criteria such as Simon Broom Register or the Dutch Lipid Clinic Network Criteria, and having no other contributing factors towards CAD such as hypertension, glucose intolerance and obesity, their elevated endothelial activation status implies that they may still be at risk of atherogenesis. However, there is limited data to further corroborate this observation.

There have been previous large prospective studies of healthy individuals showing a relationship between sICAM-1 and incidence of CAD [35–38]. Another trial reported that levels of sICAM-1 were found to be higher in healthy individuals, in whom subsequent development of symptomatic peripheral arterial disease was documented [39]. Despite lack of research on unaffected family members of FH subjects, these findings on healthy individuals can be extrapolated to infer that related unaffected family members with higher ICAM-1 levels carry similar higher risk of developing CAD as seen in this current study.

Atherosclerosis represents a chronic inflammatory state and provides a critical pathophysiological link between plaque formation and acute rupture leading to occlusion and infarction [5]. The activation of NF-κB results in the production of several proinflammatory mediators by macrophages that subsequently activate systemic release of inflammatory acute phase proteins such as hsCRP and IL-6 [40]. Expression of a spectrum of inflammatory factors, such as hsCRP and proinflammatory cytokines including interleukin-1 (IL-1) and IL-6, constitutes a strong link between inflammation and atherogenesis in CAD. In this study, hsCRP concentrations of FH index cases were significantly elevated compared to NC subjects. Interestingly, unaffected family members matched for smoking, BP, BMI, glucose levels, also have higher hsCRP concentration compared to their related FH indexed cases and normolipidaemic healthy controls. Having excluded confounding factors that could contribute to enhanced inflammation and endothelial activation, the elevated hsCRP and sICAM-1 observed among related unaffected members of FH patients could be due, in part, to the presence of mutation that may not phenotypically express in the classical manner defined by FH diagnostic criteria. Therefore, the need to consider including assessment of Inflammation and endothelial activation status during family screening may help detect family members who are still at risk of atherogenesis despite having normal cholesterol levels and absence of lipid stigmata.

Although E-selectin and sICAM-1 have been considered a strong biomarker for endothelial injury [41, 42], significant elevation of serum E-selectin concentration was not observed in RUC when comparing with NC. The possible explanation for this could be in the differences of the shedding timeline of these adhesion molecules from endothelial cells following endothelial injury. One study reported a maximal release of E-selectin into the supernatant 6–12 h after activation of human vascular endothelial cells (HUVEC) and decreased to below detection limit 24 h after activation. In contrast, release of ICAM- 1 gradually increased following activation, reaching a plateau after 24 h and remaining constant for 3 days [43] The prolonged presence of ICAM-1 molecules in circulation could have contributed to the significantly higher levels observed in the RUC compared to NC but not with E-selectin. Further studies is needed to verify, validate and determine clinical reference ranges for these analytes to establish them as biomarkers of coronary risk. In addition, a review on the role of E-selectin in cardiovascular disease highlights that the various studies comparing E-selectin in CAD-related diseases showed unequivocal results when compared to age and sex-matched healthy controls [44].

This study also reported enhanced oxidative stress in FH subjects compared to NC and related unaffected family members. Interestingly, such enhancement was also observed among family members of indexed cases as illustrated by higher MDA concentrations in RUC compared to NC. The reference range for MDA based on the International Federation of Clinical Chemists (IFCC), was established as >1.24 g/L [45], which is greatly elevated in FH and RUC, and strongly suggests heightened oxidative stress status. MDA is a reflection of lipid peroxidation and several studies have shown it to be the strongest predictor of a 3-year increase in carotid wall thickness in a regression model containing more than 30 variables [46]. Due to technical limitations, comparison of oxidative biomarkers between the three groups was restricted to oxLDL and MDA. Nevertheless, previous reports have shown that MDA determination by ELISA, which our method has been validated against, correlates with established methods for measurement of lipid peroxidation products such as MDA by HPLC and ISP by GCMS [47]. They have also shown that MDA measurement can have good inter- and intra-assay coefficients of variations [47, 48]. The finding of enhanced MDA is suggestive of increased pro-atherogenesis status of relatives of indexed cases who did not meet the diagnostic criteria for FH.

The findings of augmented inflammation, endothelial activation and oxidative stress in family members of FH subjects without classical clinical presentation of the disease as described by various FH diagnostic criteria suggest that there may be a genetic influence that may not phenotypically present as FH but carries the risk for CAD. It further suggests that perhaps a diagnostic criteria for FH, specific for Malaysian population, may need to be established to improve diagnostic accuracy, with an inclusion of genetic testing as one of its criteria. The current SB diagnostic criteria used in our centre was adopted as it is the most widely used criteria that is easily remembered and therefore more applicable in a day-to-day clinic. However, its disadvantage over the other criteria [the US MedPed Program (USMedPed), Japanese FH Criteria and Dutch Lipid Clinic Network (DLCC)] is that it may overlook a substantial proportion of FH patients, mainly those with a mild phenotype and the pediatric population in whom the phenotype has not yet emerged [49]. This could account for the underdiagnosed RUC with significantly higher biomarkers of atherogenesis compared to NC in this study. Genetic testing offers a more definitive diagnosis although it remains a costly and laborious analysis to perform in this country and therefore, not practiced. This was a limitation of this study, where due to lack of technical expertise and budget constraints, genetic determination of FH mutations was not possible.

Findings from this study underscore the relevance of 1) establishing a modified, Malaysian-based diagnostic criteria for FH that could improve the diagnostic accuracy of related family members who do not fulfill with lower cholesterol concentrations and with no classical phenotypic expression of FH, 2) conducting coronary risk stratification for family members of FH patients who either fulfil or do not fulfil the diagnostic criteria, 3) improving risk stratification strategies by including biomarkers of inflammation, endothelial activation and oxidative stress in risk scoring systems, other than the already established risk factors such as hypertension, smoking, dyslipidaemia, diabetes mellitus and family history of CAD and 4) determining as well as monitoring coronary risk targets such as blood pressure, LDL-c and HDL-c concentrations, glucose and/ or HbA1c in family members of FH patients.

## Conclusion

This present study demonstrates that patients with FH have higher inflammatory, endothelial activation and oxidative stress status compared to NC and their related unaffected family members which is in keeping with several previous studies suggesting the underlying mechanisms leading to greater risk of developing atherosclerosis-related complications such as CAD. The findings of elevated biomarkers in RUC after correcting for confounding factors, collectively, imply that they are at higher risk of CAD compared to the normal population. Therefore, although it is recommended to conduct cascade screening among family members of FH index cases, it may not sufficient to only determine and manage relatives who are FH positive without further investigating risk factors for CAD, which include biomarkers reflecting atherogenesis, in all relatives to identify those at risk for CAD despite being FH negative. Furthermore, where once related unaffected normolipaemic relatives were considered to have low risk for atherogenesis, this perception may need to be relooked as increased inflammation, endothelial activation and oxidative stress status, which are key components of atherogenesis, were observed among these subjects compared to normolipaemic unrelated controls. It would be useful to determine if these elevated biomarkers in related unaffected family members translates to the initiation and progression of atherosclerosis. Therefore future studies investigating correlations between morphological marker of early atherosclerosis such as intima media thickness (IMT) and biomarkers of atherogenesis in this cohort would further enhance our understanding of the degree of coronary risk these unaffected relatives may carry.

## Abbreviations

apoB: Apolipoprotein B
BMI: Body mass index
BP: Blood pressure
CAD: Coronary artery diseases
DBP: Diastolic blood pressure
ELISA: Enzyme linked immunorsobent assay
FCH: Familial combined hyperlipidaemia
FH: Familial hypercholesterolaemia
FPG: Fasting plasma glucose
fT4: Free thyroxine
hsCRP: High sensitivity C-Reactive Protein
IFCC: International Federation of Clinical Chemists
IL-1: Interleukin – 1
IL-6: Interleukin – 6
IMT: Intima media thickness
ISP: F2-isoprostane
LDL-c: Low density lipoprotein cholesterol
LDL-R: Low density lipoprotein – Receptor
MDA: Malondialdehyde
NC: Normolipaemic subjects
Ox-LDL: Oxidized low density lipoprotein
PCSK9: Proprotein convertase subtilisin/kexin type 9
RUC: Related unaffected relatives
SBP: Systolic blood pressure
sICAM-1: Soluble intracellular adhesion molecule – 1
sVCAM: Soluble vascular adhesion molecule
TC: Total cholesterol
TG: Triglyceride
TSH: Thyroid stimulating hormone
